# Association Between Childhood Asthma and Oral Health-Related Quality of Life in Young Children: A Cross-Sectional Study Using the SOHO-5

**DOI:** 10.3390/dj14050297

**Published:** 2026-05-13

**Authors:** Susana Valbom Morgado, João Gaspar Marques, Margarida Tejada Nunes, Ana Coelho Canta, Paula Faria Marques

**Affiliations:** 1 Unidade de Investigação em Ciências Orais e Biomédicas (UICOB), Faculdade de Medicina Dentária, Universidade de Lisboa, Rua Professora Teresa Ambrósio, 1600-277 Lisbon, Portugal; ana-coelho@edu.ulisboa.pt (A.C.C.); paulafariamarques@campus.ul.pt (P.F.M.); 2 Allergy and Clinical Immunology Department, Hospital Dona Estefânia, São José Local Health Unit, 1169-045 Lisbon, Portugal; gasparmarques@yahoo.com.br (J.G.M.); margarida.tejada@gmail.com (M.T.N.); 3 Lisbon Academic Medical Center (CAML), 1649-028 Lisbon, Portugal; 4 Comprehensive Health Research Center (CHRC), NOVA Medical School, NOVA University Lisbon, 1169-056 Lisbon, Portugal

**Keywords:** asthma, children, dental caries, salivary flow, oral health-related quality of life, SOHO-5

## Abstract

**Aim:** Asthma is a chronic condition with high prevalence in pediatric populations and may negatively influence oral health. The primary aim of this study was to evaluate the association between asthma and oral health-related quality of life (OHRQoL) in Portuguese children aged 6 to 8 years. Secondary aims included comparing caries experience, salivary parameters, and other clinical oral health indicators between asthmatic and non-asthmatic peers. **Materials and Methods:** A cross-sectional study was conducted with 89 child–parent pairs using a convenience sampling approach. Children with asthma were recruited from a hospital immunoallergology service, and healthy controls were recruited from a primary school. Data collection included parent-administered questionnaires on sociodemographic and behavioral factors, the Portuguese version of the SOHO-5 (child self-report and parent proxy forms), and standardized intraoral examinations assessing caries (WHO criteria, 5th edition), malocclusion, gingival bleeding, dental erosion, mucosal lesions, and molar–incisor hypomineralization. Stimulated salivary flow was measured. Bivariate statistical analyses and multivariable regression models were performed using SPSS (v.29), with a significance level set at *p* < 0.05. **Results:** Asthmatic children had significantly higher caries prevalence in both primary (52.6% vs. 27.5%, *p* = 0.027) and permanent dentition (32.4% vs. 0%, *p* < 0.001), as well as higher mean dmft scores (2.68 vs. 1.14, *p* = 0.026), reduced stimulated salivary flow (78.9% vs. 41.2% with low flow, *p* < 0.001), and worse child-reported SOHO-5 scores (mean 2.42 vs. 1.25, *p* = 0.004). After multivariable adjustment, asthma remained a significant independent predictor of low salivary flow (OR = 4.017, 95% CI: 1.443–11.178, *p* = 0.008), while the association with caries was attenuated and no longer significant (OR = 1.345, *p* = 0.590). Pain experience in the past year was the strongest predictor of OHRQoL across all multivariable models (SOHO-5 child: B = 1.583, *p* = 0.006; SOHO-5 total: B = 4.970, *p* < 0.001), indicating that children with pain history reported substantially worse OHRQoL. After adjustment, asthma did not reach statistical significance for either child-reported (B = 0.732, *p* = 0.090) or total OHRQoL scores (B = 0.693, *p* = 0.293). These findings should be interpreted cautiously given the limited number of covariates included in the models, constrained by the available sample size. **Conclusions:** Within the limitations of this cross-sectional study, including a small and non-probabilistic sample, asthmatic children presented a higher caries burden and a markedly higher prevalence of low stimulated salivary flow compared with non-asthmatic peers. Asthma remained a significant independent predictor of low salivary flow after multivariable adjustment, while the association with caries was attenuated, suggesting partial confounding by dietary habits. These findings highlight the importance of integrating oral health surveillance into the routine care of asthmatic children, with particular attention paid to salivary function and caries prevention.

## 1. Introduction

Oral health is a fundamental determinant of overall health and an important contributor to quality of life, as it influences essential daily functions such as speaking, eating, smiling and contributes significantly to children’s social and emotional development [1]. Traditional clinical indicators of oral disease, or “normative” assessments, provide limited insight into the broader consequences of oral conditions on children’s lives [2,3]. To address this limitation, oral health-related quality of life (OHRQoL) has been extensively used as a multidimensional measure of the impact of oral diseases and disorders on individuals and communities [3]. Unlike normative assessments, OHRQoL instruments capture subjective experiences and the psychosocial consequences of oral conditions, reflecting patients’ perspectives and their families [3]. Such measures are also useful as outcomes in evaluating dental interventions and for identifying patient-centered needs [3].

Health-related quality of life (HRQoL) indices have been increasingly integrated into disease surveillance systems [4], enabling morbidity comparisons and guiding health policy priorities. Dental caries, despite significant advances in prevention and treatment, remains one of the most prevalent chronic diseases of childhood. Globally, an estimated 532 million children have untreated caries in primary teeth [5]. Gingivitis is also highly prevalent, with severity and extent increasing with age, peaking during puberty before a modest decline in adolescence [6,7]. Malocclusion is another frequent condition, with several epidemiological studies reporting high prevalence and growing demand for orthodontic care among children [7,8]. In addition to behavioral and individual factors, social determinants play a key role in shaping the prevalence and severity of oral diseases. Caries represents not only a health condition but also a social problem, disproportionately affecting socioeconomically disadvantaged populations [3]. Children from families with lower socioeconomic status tend to have poorer OHRQoL, ref. [9], reflecting both reduced capacity to access and respond to oral health problems and differences in perception of their health needs.

Asthma is a common chronic respiratory disease, with a prevalence ranging between 1% and 29% worldwide [10]. In Portugal, data from the INAsma survey report an estimated prevalence of 8.4% among children [11]. Beyond respiratory symptoms, asthma impacts daily functioning, psychological well-being, and overall quality of life for children and their families [12].

Several studies have examined the potential impact of asthma and its pharmacological management on the oral health of children and adolescents [13]. Asthmatic individuals frequently present with oral mucosal dehydration, reduced salivary flow, and lower oral pH, often associated with habitual mouth breathing, a common characteristic of this condition, as well as with the side effects of medications that diminish salivary secretion and alter salivary composition [14,15]. Moreover, some asthma medications, contain sugar further increasing susceptibility to oral health problems. The reported oral findings include increased incidence of dental caries, tooth wear, gingivitis, and an altered salivary composition and flow rate [16,17].

Asthma and its treatment may influence oral health through multiple pathways, including reduced salivary flow, altered oral pH, and mouth breathing. These changes may increase susceptibility to dental caries and oral discomfort, which in turn may affect daily functions such as eating and speaking, ultimately impacting OHRQoL. Nevertheless, the available evidence remains inconsistent and potentially confounded by factors such as socioeconomic status, dietary patterns, and methodological heterogeneity across studies [16,17]. While several investigations have focused on the clinical oral health of children with asthma, relatively few have examined its association with oral health–related quality of life (OHRQoL). Evidence addressing this relationship in Portuguese pediatric populations is scarce. Therefore, the present study was designed to address this gap by investigating OHRQoL and clinical oral health indicators in Portuguese children aged 6 to 8 years with and without asthma, using the validated Portuguese version of the SOHO-5. To our knowledge, this is the first study to specifically examine the association between asthma and OHRQoL in this age group using a child self-report instrument in a Portuguese population.

The primary aim of this study was to evaluate oral health-related quality of life (OHRQoL) in Portuguese children with asthma compared with non-asthmatic peers, using the SOHO-5 instrument. Secondary aims included comparing caries experience, salivary parameters, gingival health, and other clinical oral health indicators between the two groups.

## 2. Materials and Methods

This cross-sectional study was approved by the Ethics Committee of the Faculty of Dental Medicine, University of Lisbon (reference: 202326) and Ethics Committee of São José Local Health Unit (1512/2024). The study population consisted of children aged 6 to 8 years and their parents, recruited from the Lisbon metropolitan area. Both children with asthma and healthy controls were included.

### 2.1. Sample Size Determination

An a priori sample size calculation was performed using G*Power software v.3.1 (Heinrich-Heine-Universität Düsseldorf, Düsseldorf, Germany). Parameters included a significance level (α) of 0.05, statistical power (1 − β) of 0.80, and a large effect size (Cohen’s d = 0.8), assumed in the absence of specific prior data on OHRQoL differences between asthmatic and non-asthmatic children in this age group and context. The analysis indicated that a minimum of 26 children per group would be required for the primary group comparison. It should be noted that this calculation was not designed to support subgroup or stratified analyses.

### 2.2. Recruitment and Eligibility

A non-probabilistic convenience sampling strategy was employed. Children clinically diagnosed with asthma and undergoing maintenance therapy with inhaled corticosteroids (ICS), with or without additional controller medications, were recruited from the Immunoallergology Service of D. Estefânia Children’s Hospital. Healthy controls were recruited from a primary school in Lisbon. Eligibility criteria for both groups were:

- Age 6 to 8 years;
- Absence of other chronic illnesses;
- Not wearing orthodontic appliances;
- No systemic antibiotic use in the preceding two weeks.

Written informed consent was obtained from all parents, and verbal assent was obtained from the children.

### 2.3. Asthma Treatment Characterization

For each child in the asthma group, the total daily dose of inhaled corticosteroids was recorded and classified as low, medium, or high according to the Global Initiative for Asthma (GINA) guidelines [18]. This categorization enabled a standardized evaluation of treatment intensity and allowed for the investigation of potential dose–response relationships with oral health–related outcomes.

### 2.4. Data Collection

Data were collected between June 2024 and July 2025. All clinical examinations were conducted by a single trained examiner, assisted by a recorder, ensuring consistency across assessments. Intraexaminer reliability was assessed in a subsample of 17 children. Cohen’s kappa coefficient for caries in primary teeth was 0.850, indicating very good agreement.

Child assessment: Children completed the Portuguese child self-report version of the SOHO-5 questionnaire [19] and underwent intraoral examination.

Intraoral examination: Before clinical assessment, tooth surfaces were cleaned with gauze to facilitate visualization. Clinical assessments recorded the presence of dental caries (WHO criteria), incisor-molar hypomineralisation, dental erosion, oral mucosal lesions, malocclusion, spontaneous gingival bleeding, and visible bacterial plaque (Oral Hygiene Index-Simplified). Gingival bleeding was assessed at six sites per tooth (mesiobuccal, buccal, distobuccal, mesiolingual, lingual, and distolingual) using a periodontal probe with a ball-shaped tip, following the Bleeding on Probing (BOP) method as recommended by the World Health Organization [20]. In addition, stimulated saliva was collected over five minutes by having the child chew on a piece of sterile paraffin wax, and the volume was measured to assess salivary flow rate. A stimulated salivary flow rate below 1.0 mL/min was classified as low, based on established reference values for this age group [21].

Parent questionnaire: A self-administered questionnaire collected sociodemographic information (age, sex, nationality, siblings, parental education), oral health behaviors (brushing frequency, fluoride toothpaste use, supervision, cariogenic food intake, previous dental visits), early-life health information, and the Portuguese parent version of the SOHO-5 [19].

The clinical examination was conducted by a trained examiner, assisted by a recorder. Instruments included a mouth mirror (ASA reference 2200E-5, 24 mm diameter) and a periodontal probe with a ball-shaped tip (ASA reference 0702L-12S), following World Health Organization recommendations [20]. Illumination was provided by a white LED headlight. All procedures adhered to standard infection control protocols, including sterilization of instruments and the use of gloves and masks.

### 2.5. SOHO-5 Instrument

The SOHO-5 comprises two versions: a child self-report and a parent proxy-report, each containing 7 items. The child version covers difficulties in eating, drinking, speaking, playing, sleeping, and avoiding smiling due to pain or appearance concerns. Items are rated on a 3-point scale (0 = no; 1 = a little; 2 = a lot). The parent version assesses similar domains, with the addition of self-confidence, rated on a 5-point scale (0 = not at all to 4 = a great deal, plus “don’t know”). Questionnaires with more than two “don’t know” responses were excluded.

Scores range from 0 to 14 for the child version and 0 to 28 for the parent version, with higher scores indicating poorer OHRQoL [22]. The validated and culturally adapted Portuguese version of the SOHO-5 used in this study was adapted from the Brazilian Portuguese version [23], with minor linguistic modifications to ensure cultural appropriateness [19]. Content validity was confirmed by an expert panel of three dentists (two Portuguese, one Brazilian) with relevant research experience. Psychometric analysis demonstrated good internal consistency, with Cronbach’s alpha values of 0.86 (child version), 0.83 (caregiver version), and 0.84 (total score), indicating high reliability [19]. Item-total correlations were moderate, with no items requiring exclusion. Test–retest reliability was assessed using the Intraclass Correlation Coefficient (ICC), yielding values of 0.82 for the child version and 0.95 for the total score, indicating good to excellent temporal reliability [19].

### 2.6. Statistical Analysis

Data were analyzed using SPSS, version 29 (IBM Corp., Armonk, NY, USA). Descriptive statistics were calculated for all variables. Continuous variables were described using mean and standard deviation (SD), median and interquartile range (Q1–Q3) when data were not normally distributed. Categorical variables were expressed as absolute frequencies and percentages.

Normality of distribution was assessed using the Kolmogorov–Smirnov test. For group comparisons, the Chi-square test and Fisher’s Exact test were used for categorical variables, and the Mann–Whitney-U test was used for continuous non-normally distributed variables. Subgroup analyses were conducted within the asthma group to explore associations between clinical variables and OHRQoL scores.

To further examine the independent association between asthma and oral health outcomes, multivariable regression analyses were performed. Binary logistic regression was used for dichotomous outcomes (caries in primary teeth and low stimulated salivary flow), and linear regression for continuous outcomes (SOHO-5 child and total scores). To select covariates for inclusion in the final models, preliminary separate regression analyses were conducted examining sociodemographic, behavioral, and asthma-related variables as potential predictors of each outcome. Given the limited sample size and the need to maintain adequate event-per-variable ratios, the number of covariates in each final model was restricted to a maximum of two, in addition to the main exposure variable (presence of asthma). Based on this approach, pain experience in the past year and frequency of sugary drinks consumption were selected as covariates across all final models. Model fit for logistic regression models was assessed using the Hosmer–Lemeshow test and Nagelkerke R^2^, and for linear regression models using the F-test and R^2^.

A significance level of *p* < 0.05 was applied to all analyses.

## 3. Results

The final sample comprised 89 child–parent pairs. Children had a mean age of 7.3 years (SD = 0.8); 57.7% were male, and 88.8% lived with both parents. University-level education was reported by 56% of mothers and 54% of fathers. Among the 38 children with asthma, 7.9% (*n* = 3) were treated with low doses of ICS, 52.6% (*n* = 20) medium doses of ICS, and 39.5% (*n* = 15) with high doses of ICS. Regarding oral health behaviors, 75.3% of children attended regular dental check-ups, and 70.8% had visited a dentist within the previous six months. Dental pain in the past year was reported by 18%. Brushing twice daily was reported by 84.3%, use of fluoridated toothpaste by 83.1%, and daily flossing by 14.6%. Supervised brushing was reported by 61.8% of participants. Weekly consumption of sugary drinks and cariogenic foods was reported by 65.2% and 73%, respectively.

The prevalence of caries in deciduous dentition was 38.2% (*n* = 34), with a mean dmft score of 1.8 (SD = 2.75). In permanent teeth, caries prevalence was 14% (*n* = 12), with a mean DMFT of 0.35 (SD = 0.90). Other clinical findings included gingival bleeding (9%), molar–incisor hypomineralization (28.1%), and mild dental erosion (3.4%). Aphthous ulcers (4.5%) were the most common mucosal lesion. Malocclusion was also frequent: 48.7% presented a Class II molar relationship, 5.3% Class III, 14.6% crossbite, 16.9% open bite, and 15.7% deep bite. Stimulated salivary flow was <1.0 mL/min in 57.3% of children (Table 1).

### 3.1. Group Comparisons

Caries experience in the primary dentition was significantly higher among asthmatic children (52.6%) compared with non-asthmatic children (27.5%) (*p* = 0.027). Notably, caries in permanent dentition occurred exclusively in the asthma group (32.4%), with no cases among non-asthmatic children (*p* < 0.001) (Table 2). Although the absence of caries in permanent teeth among controls may partly reflect the young age of the sample—in which permanent dentition is not yet fully erupted—the difference between groups remained statistically significant and warrants attention.

The mean dmft was significantly higher in asthmatic children (2.68 ± 3.27) compared with controls (1.14 ± 2.08; *p* = 0.026). Asthmatic children also showed higher numbers of decayed primary teeth (2.05 ± 2.71 vs. 0.67 ± 1.63; *p* = 0.003) and missing primary teeth (0.37 ± 0.88 vs. 0.02 ± 0.14; *p* = 0.007). In the permanent dentition, asthmatic children had a significantly higher number of decayed teeth (0.57 ± 1.07; *p* < 0.001) and greater mean DMFT scores (0.73 ± 1.24; *p*< 0.001). No significant differences were observed between groups for gingival bleeding, molar–incisor hypomineralization, dental erosion, or oral mucosal alterations (*p* > 0.05). Similarly, no differences were found in sagittal, transversal, or vertical malocclusion patterns (*p* > 0.05).

Stimulated salivary flow < 1.0 mL/min was significantly more prevalent among asthmatic children (78.9%) compared with controls (41.2%; *p* < 0.001) (Table 3).

### 3.2. SOHO-5 Outcomes

In the study sample (*n* = 89), the child-reported score ranged from 0 to 9 (median 1.00 [Q1 = 0.00; Q3 = 3.00]; mean 1.75, SD = 1.98), the parent-reported score ranged from 0 to 14 (median 0.00 [Q1 = 0.00; Q3 = 1.00]; mean 1.20, SD = 2.58), and the total score ranged from 0 to 14 (median 2.00 [Q1 = 0.00; Q3 = 4.00]; mean 2.94, SD = 3.48). Asthmatic children consistently reported poorer OHRQoL compared with non-asthmatic peers across all three scores, reaching statistical significance for child-reported (*p* = 0.004) and total scores (*p* = 0.001), but not for parent-reported scores (*p* = 0.059) (Table 4).

Although the absolute SOHO-5 scores were generally low in both groups—suggesting an overall favorable oral health-related quality of life—the statistically significant differences between groups indicate that asthmatic children experience a meaningfully greater burden in their daily oral health experiences, particularly in functional domains such as eating and speaking. Functional domains were most affected: “difficulty eating” and “difficulty speaking” in the child version, and “difficulty eating” and “difficulty sleeping” in the parent version. In the child-reported SOHO-5, the response ‘No’ was the most frequently endorsed across all items.

Significant differences were observed, particularly among 8-year-olds, where parent-reported and total scores were higher in the asthma group. Higher SOHO-5 scores were also associated with parental higher education and with the presence of caries. Dental pain was strongly associated with worse OHRQoL in both groups. Subgroup analysis revealed that asthmatic children with low salivary flow reported significantly poorer OHRQoL on both child-reported (*p* = 0.009) and total scores (*p* = 0.005), although parent-reported differences were not significant (*p* = 0.104). (Table 5 and Table 6) These subgroup analyses were not pre-specified in the original study design and should therefore be interpreted as exploratory. Given the small cell sizes and the increased risk of type II error, no definitive conclusions can be drawn from these findings.

Two binary logistic regression models were fitted for caries in primary teeth (EPV = 11.3) and low stimulated salivary flow (EPV = 12.7), and two linear regression models for SOHO-5 child and total scores. All models showed adequate fit. For caries in primary teeth, the logistic model explained approximately 38% of the variance (Nagelkerke R^2^ = 0.380; Hosmer–Lemeshow: χ^2^ = 4.321, *p* = 0.504). After adjustment, asthma was not a significant independent predictor (OR = 1.345, 95% CI: 0.457–3.958, *p* = 0.590), suggesting that the bivariate association may be partially confounded by dietary habits. Frequency of sugary drinks consumption was a significant predictor of higher caries odds (OR = 3.994, 95% CI: 1.357–11.753, *p* = 0.012). Pain experience in the past year was also a significant predictor of higher caries odds (OR = 14.250, 95% CI: 2.543–79.849, *p* = 0.003), likely reflecting greater dental disease burden in symptomatic children. For low salivary flow, the model explained approximately 28% of the variance (Nagelkerke R^2^ = 0.277; Hosmer–Lemeshow: χ^2^ = 5.088, *p* = 0.278). In contrast to caries, asthma remained a significant independent predictor of low salivary flow after adjustment (OR = 4.017, 95% CI: 1.443–11.178, *p* = 0.008), confirming that this association is not explained by behavioral confounders. Pain experience was also significant (OR = 10.907, 95% CI: 1.230–96.746, *p* = 0.032).

For OHRQoL outcomes, both linear models were statistically significant (SOHO-5 child: F(3,85) = 5.633, *p* = 0.001, R^2^ = 0.166; SOHO-5 total: F(3,85) = 15.888, *p* < 0.001, R^2^ = 0.359). Pain experience in the past year was the strongest predictor across both models (SOHO-5 child: B = 1.583, 95% CI: 0.473–2.693, *p* = 0.006; SOHO-5 total: B = 4.970, 95% CI: 3.266–6.673, *p* < 0.001), indicating that children with pain history reported substantially worse OHRQoL. Frequency of sugary drinks was not significant in either model (*p* = 0.845; *p* = 0.882). After adjustment, asthma showed a borderline non-significant trend towards poorer child-reported OHRQoL (B = 0.732, 95% CI: −0.116 to 1.581, *p* = 0.090) and was not a significant predictor of total OHRQoL (B = 0.693, 95% CI: −0.609 to 1.996, *p* = 0.293). Detailed results are presented in Table 7.

## 4. Discussion

This study examined the association between childhood asthma and oral health outcomes, including caries experience, salivary flow, and oral health-related quality of life (OHRQoL). The findings suggest that children with asthma may be at higher risk of certain oral health problems, including higher caries prevalence, higher dmft/DMFT indices, and reduced stimulated salivary flow. These clinical differences were associated with poorer OHRQoL, particularly from the child’s perspective. However, these associations should be interpreted with caution given the cross-sectional design, the convenience sampling strategy, and the differences in socioeconomic profile between groups observed in this study.

The higher caries prevalence observed among asthmatic children in the bivariate analysis is consistent with previous reports. Meta-analyses by Alavaikko et al. and Agostini et al. have reported an approximately 1.5-fold increased risk of caries in children with asthma [24,25]. However, the multivariable analysis conducted in the present study suggests that this association may be partially confounded by dietary habits—specifically, frequency of sugary drinks consumption—rather than reflecting a direct effect of asthma per se (OR = 1.345, *p* = 0.590 after adjustment). This finding is consistent with evidence highlighting the role of behavioral and socioeconomic confounders in the relationship between asthma and caries [17] and underscores the need for caution when interpreting bivariate associations in this context.

The markedly higher prevalence of low salivary flow among asthmatic children, and its persistence as a significant independent predictor after multivariable adjustment (OR = 4.017, 95% CI: 1.443–11.178, *p* = 0.008), suggests that asthma and its pharmacological management may compromise salivary gland function through mechanisms that are not explained by behavioral factors alone [26]. Kargul et al. similarly reported decreased salivary and plaque pH in asthmatic children using inhalers, further supporting the potential role of reduced salivary protection in this population [27].

Although overall OHRQoL scores were generally low in both groups, suggesting a relatively favorable oral health-related quality of life, asthmatic children consistently reported higher SOHO-5 scores compared with non-asthmatic peers in the bivariate analysis. These differences were most pronounced in functional domains such as eating and speaking, which may reflect the combined burden of oral and respiratory symptoms on daily functioning. Comparable scores were observed in a study conducted in a healthy school population, where mean total SOHO-5 scores were 1.86 ± 2.27 for child self-report and 2.65 ± 3.13 for parental report [28], suggesting that the scores observed in our control group are consistent with population norms. By contrast, Abanto et al. reported higher mean scores in both child (3.32 ± 3.22) and parent versions (5.18 ± 6.28) in a Brazilian school population [29], highlighting the variability in OHRQoL scores across different populations and healthcare contexts.

After multivariable adjustment, however, the association between asthma and OHRQoL was attenuated and did not reach statistical significance, either for child-reported (B = 0.732, *p* = 0.090) or total scores (B = 0.693, *p* = 0.293). This suggests that the observed bivariate differences in OHRQoL may be partly explained by confounders such as pain experience and dietary habits rather than by asthma alone. The discrepancy between child and parent-reported scores is also noteworthy: parents consistently underestimated the oral health burden compared with their children’s self-assessments, reinforcing the importance of incorporating child-reported outcomes in both research and clinical practice [30]. Reliance on parental proxy reports alone may therefore mask the true impact of oral conditions on children’s daily lives.

Dental pain emerged as the strongest predictor of OHRQoL across all multivariable models (SOHO-5 child: B = −1.583, *p* = 0.006; SOHO-5 total: B = −4.970, *p* < 0.001), in some cases outweighing the contribution of asthma itself. This finding underscores the importance of preventive strategies and timely management of dental caries in all children, and particularly in those with chronic respiratory conditions such as asthma, who may face additional barriers to oral healthcare access. Subgroup analyses further suggested that asthmatic children with additional risk factors—namely caries in permanent teeth and low salivary flow—reported markedly poorer OHRQoL, although these findings should be interpreted with caution given the small cell sizes involved.

### Strengths and Limitations

Several limitations should be acknowledged. The cross-sectional design precludes causal inference, and the relatively small sample size, though justified by an a priori power analysis, may have limited the detection of more subtle associations, particularly in subgroup analyses; therefore, stratified findings should be interpreted with caution and considered exploratory. The non-probabilistic convenience sampling from two different settings, a hospital immunoallergology service and a primary school, may introduce systematic differences in socioeconomic status and healthcare utilization between groups, which may limit the comparability between groups.

Notably, significant differences in parental education were observed between groups, suggesting that socioeconomic confounding may not have been fully addressed despite the multivariable adjustment conducted, and residual confounding cannot be excluded. The multivariable analyses were also constrained by the available sample size, limiting the number of covariates that could be included while maintaining adequate event-per-variable ratios. Additionally, parental reports of oral health behaviors may be subject to recall or social desirability bias. Finally, dietary habits were only partially assessed.

Despite these limitations, this study provides novel insights into an important but underexplored area. To our knowledge, it is the first study to specifically examine the association between asthma and OHRQoL in children aged 6 to 8 years using a child self-report instrument in a Portuguese population. By focusing on this developmental stage, when both primary and permanent dentition coexist, the study captures a critical window of vulnerability for oral health. The asthma diagnosis was established by pediatric immunoallergologists with clinical experience, ensuring diagnostic accuracy. The use of a validated and culturally adapted Portuguese version of the SOHO-5, with demonstrated internal consistency (Cronbach’s α = 0.86) and temporal reliability (ICC = 0.82), ensures methodological rigor and comparability with international research. The integration of both clinical indicators and child-reported quality-of-life assessments, together with the inclusion of multivariable analyses, represents a key methodological strength of this study. The inclusion of pain experience as a covariate in multivariable models represents an additional methodological strength, as this variable emerged as the dominant predictor of OHRQoL and its omission could have led to overestimation of the effect of asthma.

The findings of this study highlight several directions for future research. First, longitudinal studies with larger and matched samples are needed to establish causal pathways between asthma, its pharmacological management, and oral health outcomes, as well as to assess changes over time in both salivary function and OHRQoL. Second, future studies should incorporate comprehensive dietary assessment, including sugar intake frequency and quantity, to better disentangle the contributions of asthma and behavioral risk factors to caries experience in this population. Third, matched study designs or multivariable approaches with adequate sample sizes would allow for more robust control of socioeconomic confounders, particularly parental education and healthcare access.

Finally, given that low salivary flow was the only oral health outcome for which asthma remained a significant independent predictor after multivariable adjustment, future studies should specifically investigate the role of inhaled corticosteroids and bronchodilators on salivary gland function, including objective measures of salivary composition and buffering capacity.

## 5. Conclusions

Within the limitations of this cross-sectional study, children with asthma exhibited a higher caries burden and a significantly higher prevalence of low stimulated salivary flow compared with non-asthmatic peers. After multivariable adjustment, asthma remained a significant independent predictor of low salivary flow, while the association with caries was attenuated and no longer statistically significant, suggesting partial confounding by dietary habits. Dental pain emerged as the primary determinant of OHRQoL, and child-reported outcomes consistently captured a greater burden than parental proxy reports. These findings underscore the importance of integrating oral health surveillance into the routine care of asthmatic children, with particular attention paid to salivary function and caries prevention.

## Figures and Tables

**Table 1:** Sociodemographic, oral health behavior, and clinical characteristics of the study population (*n* = 89).

Variable		*n*	%
Age	6	19	21.3%
	7	21	23.6%
	8	49	55.1%
Sex	Female	38	42.7%
	Male	51	57.3%
Mother Education	Basic education or less	7	7.9%
	Secondary education complete	26	29.2%
	High education complete	56	62.9%
Father Education	Basic education or less	6	6.7%
	Secondary education complete	29	32.6%
	High education complete	54	60.7%
Frequent dental appointments	Never	7	7.9%
	Symptomatic dental attenders	15	16.9%
	Regular attenders, even in the absence of complaints	67	75.3%
Pain reported over the past year	Yes	16	18.0%
	No	73	82.0%
Daily tooth brushing frequency	No tooth brushing	1	1.1%
	Once a day	13	14.6%
	Twice a day	75	84.3%
Daily fluoride toothpaste use	Yes	74	83.1%
	No	15	16.9%
Daily use of dental floss	Yes	13	14.6%
	No	76	85.4%
Supervised brushing	Yes	55	61.8%
	No	34	38.2%
Frequency of consumption of sweets and other cariogenic foods	Never	5	5.6%
	1/2 times per week	65	73.0%
	Every day	19	21.3%
Presence of caries in primary teeth	No	55	61.8%
	Yes	34	38.2%
Presence of caries in permanent teeth	No	74	86.0%
	Yes	12	14.0%
Bleeding on probing	No	81	91.0%
	Yes	8	9.0%
Molar Incisor Hypomineralization	No	64	71.9%
	Yes	25	28.1%
Sagittal malocclusion	Class I	35	46.1%
	Class II	37	48.7%
	Class III	4	5.3%
Sagittal malocclusion in deciduous dentition	Straight terminal step	8	61.5%
	Distal step	4	30.8%
	Mesial step	1	7.7%
Transversal malocclusion	Absence of transversal discrepancies	76	85.4%
	Posterior crossbite	13	14.6%
	Scissor bite	0	0.0%
Vertical malocclusion	Absence of vertical discrepancies	60	67.4%
	Open bite	15	16.9%
	Deep bite	14	15.7%
Stimulated salivary flow rate	Low rate < 1 mL/min	51	57.3%
	Normal rate > 1 mL/min	38	42.7%

**Table 2:** Caries prevalence according to asthma status in the study population.

		Presence of Asthma		
		No *n* (%) *n* = 51 (57.3%)	Yes *n* (%) *n* = 38 (42.7%)	*p*
Presence of caries in primary teeth	Yes	14 (27.5%)	20 (52.6%)	**0.027 ^1^**
	No	37 (72.5%)	18 (47.4%)	
Presence of caries in permanent teeth	Yes	0 (0%)	12 (32.4%)	**<0.001 ^1^**
	No	49 (100%)	25 (67.6%)	

*p* values in **bold** are statistically significant; ^1^ Chi-square Test.

**Table 3:** Sociodemographic, behavioral and clinical characteristics of the study population.

		Presence of Asthma		
		No *n* (%) *n* = 51 (57.3%)	Yes *n* (%) *n* = 38 (42.7%)	*p*
Age	6	6 (11.8%)	13 (34.2%)	**0.043 ^1^**
	7	13 (25.5%)	8 (21.1%)	
	8	32 (62.7%)	17 (44.7%)	
Mother Education	Basic education or less	1 (2%)	6 (15.8%)	**<0.001 ^2^**
	Secondary education complete	5 (9.8%)	21 (55.3%)	
	High education complete	45 (88.2%)	11 (28.9%)	
Father Education	Basic education or less	2 (3.9%)	4 (10.5%)	**<0.001 ^2^**
	Secondary education complete	3 (5.9%)	26 (68.4%)	
	High education complete	46 (90.2%)	8 (21.1%)	
Frequent dental appointments	Never	1 (2%)	6 (15.8%)	**<0.003 ^2^**
	Symptomatic dental attenders	5 (9.8%)	10 (26.3%)	
	Regular attenders, even in the absence of complaints	45 (88.2%)	22 (57.9%)	
Pain reported over the past year	Yes	3 (5.9%)	13 (34.2%)	**<0.001 ^1^**
	No	48 (94.1%)	25 (65.8%)	
Stimulated salivary flow rate	Low rate < 1 mL/min	21 (41.2%)	30 (78.9%)	**<0.001 ^1^**
	Normal rate > 1 mL/min	30 (58.8%)	8 (21.1%)	

*p* values in **bold** are statistically significant; ^1^ Chi-square Test; ^2^ Fisher’s Exact Test.

**Table 4:** Distribution of SOHO-5 scores according to Asthma status.

	Total Population *n* = 89 (100%)	No Asthma *n* = 51 (57.3%)	Asthma *n* = 38 (42.7%)	*p*
SOHO-5 CHILD				
Median [Q1; Q3]	1.00 [0.00; 3.00]	1.00 [0.00; 2.00]	2.00 [0.75; 4.00]	**0.004 ^1^**
Mean (SD)	1.75 (1.98)	1.25 (1.70)	2.42 (2.16)	
[min; max]	[0; 9]	[0; 7]	[0; 9]	
SOHO-5 PARENT				
Median [Q1; Q3]	0.00 [0.00; 1.00]	0.00 [0.00; 1.00]	0.00 [0.00; 2.00]	0.059 ^1^
Mean (SD)	1.20 (2.58)	0.80 (2.22)	1.74 (2.94)	
[min; max]	[0; 14]	[0; 14]	[0; 11]	
SOHO-5 TOTAL				
Median [Q1; Q3]	2.00 [0.00; 4.00]	1.00 [0.00; 3.00]	3.00 [1.00; 5.75]	**0.001 ^1^**
Mean (SD)	2.94 (3.48)	2.04 (2.87)	4.16 (3.87)	
[min; max]	[0; 14]	[0; 14]	[0; 14]	

*p* values in **bold** are statistically significant; ^1^ Mann–Whitney-U test.

**Table 5:** Association of sociodemographic and behavioral variables with SOHO-5 scores, stratified by asthma group.

		Presence of Asthma	SOHO-5 CHILD		SOHO-5 PARENT		SOHO-5 Total	
			Mean (SD)	*p*	Mean (SD)	*p*	Mean (SD)	*p*
Age	6 years	Yes	3.08 (3.01)	0.05 ^1^	1.85 (2.48)	0.89 ^1^	4.92 (4.07)	0.24 ^1^
		No	0.17 (0.41)		3.33 (5.75)		3.50 (5.86)	
	7 years	Yes	1.63 (1.51)	0.24 ^1^	1.00 (2.83)	0.46 ^1^	2.63 (3.34)	0.37 ^1^
		No	1.00 (2.00)		0.69 (1.18)		1.62 (2.99)	
	8 years	Yes	3.67 (3.20)	0.08 ^1^	2.17 (4.40)	**0.02 ^1^**	4.29 (3.95)	**0.01 ^1^**
		No	1.56 (1.64)		0.38 (0.79)		1.94 (1.95)	
Mother Education	Basic or less	Yes	3.67 (3.20)	0.28 ^1^	2.17 (4.40)	0.28 ^1^	5.83 (5.30)	0.28 ^1^
		No	0.00		14.00		14.00	
	Secondary complete	Yes	2.05 (1.66)	0.48 ^1^	1.43 (2.18)	0.71 ^1^	3.48 (2.82)	0.31 ^1^
		No	1.40 (1.34)		0.60 (0.89)		2.00 (1.58)	
	High education	Yes	2.45 (2.34)	0.11 ^1^	2.09 (3.51)	**0.04 ^1^**	4.55 (4.72)	**0.04 ^1^**
		No	1.27 (1.75)		0.53 (1.22)		1.78 (2.41)	
Father Education	Basic or less	Yes	2.75 (2.36)	0.13 ^1^	3.23 (5.25)	0.62 ^1^	6.00 (6.27)	0.81 ^1^
		No	0.00		7.00 (9.89)		7.00 (9.89)	
	Secondary complete	Yes	2.23 (2.21)	0.76 ^1^	1.12 (2.03)	0.92 ^1^	3.35 (2.99)	0.70 ^1^
		No	1.67 (1.53)		0.67 (1.15)		2.33 (2.52)	
	High education	Yes	2.88 (2.10)	**0.02 ^1^**	3.00 (3.82)	**0.01 ^1^**	5.88 (4.73)	**0.003 ^1^**
		No	1.25 (1.70)		0.80 (2.22)		2.04 (2.87)	
Dental appointments	Never	Yes	1.67 (1.03)	0.57 ^1^	0.17 (0.41)	1.00 ^1^	1.83 (1.17)	0.57 ^1^
		No	1.00		0.00		1.00	
	Symptomatic dental attenders	Yes	1.40 (1.90)	0.86 ^1^	2.60 (3.81)	0.44 ^1^	4.00 (4.94)	1.00 ^1^
		No	2.00 (2.92)		1.40 (2.61)		3.40 (3.36)	
	Regular attenders	Yes	3.09 (2.31)	**0.00 ^1^**	1.77 (2.81)	0.74 ^1^	4.86 (3.67)	**0.00 ^1^**
		No	1.18 (1.56)		0.76 (2.22)		1.91 (2.84)	
Pain past year	Yes	Yes	3.31 (2.29)	1.00 ^1^	3.54 (3.86)	0.61 ^1^	6.85 (4.18)	0.36 ^1^
		No	3.33 (3.51)		6.00 (7.21)		9.33 (5.69)	
	No	Yes	1.96 (1.99)	0.07 ^1^	0.80 (1.78)	0.50 ^1^	2.76 (2.89)	0.05 ^1^
		No	1.13 (1.50)		0.48 (1.09)		1.58 (1.94)	

*p* values in **bold** are statistically significant; ^1^ Mann–Whitney-U test.

**Table 6:** Association of oral disease variables with SOHO-5 scores, stratified by asthma group.

		Presence of Asthma	SOHO-5 CHILD		SOHO-5 PARENT		SOHO-5 Total	
			Mean (SD)	*p*	Mean (SD)	*p*	Mean (SD)	*p*
Caries primary teeth	Yes	Yes	2.95 (2.37)	0.31 ^1^	2.55 (3.22)	0.17 ^1^	5.50 (4.06)	0.18 ^1^
		No	2.21 (2.49)		1.57 (3.76)		3.79 (4.28)	
	No	Yes	1.83 (1.79)	0.07 ^1^	0.83 (2.36)	0.82 ^1^	2.67 (3.11)	0.07 ^1^
		No	0.89 (1.13)		0.51 (1.19)		1.38 (1.78)	
Caries permanent teeth	Yes	Yes	2.00 (1.28)	-	2.92 (3.92)	-	4.92 (4.60)	-
		No	-		-		-	
	No	Yes	2.64 (2.51)	**0.02 ^1^**	1.24 (2.28)	0.17 ^1^	3.38 (3.57)	**0.009 ^1^**
		No	1.29 (1.72)		0.71 (2.13)		1.98 (2.82)	
Salivary flow rate	Low < 1 mL/min	Yes	2.53 (2.27)	**0.009 ^1^**	1.90 (3.00)	0.10 ^1^	4.43 (3.85)	**0.005 ^1^**
		No	1.00 (1.14)		1.05 (3.06)		2.00 (3.07)	
	Normal > 1 mL/min	Yes	2.00 (1.77)	0.28 ^1^	1.13 (2.80)	1.00 ^1^	3.13 (4.02)	0.47 ^1^
		No	1.43 (1.99)		0.63 (1.40)		2.07 (2.78)	

*p* values in **bold** are statistically significant; ^1^ Mann–Whitney-U test.

**Table 7:** Multivariable regression analyses for oral health outcomes (*n* = 89).

A. Binary logistic regression—Caries in primary teeth (EPV = 11.3; Nagelkerke R^2^ = 0.380; Hosmer–Lemeshow *p* = 0.504)				
Variable	OR	95% CI	*p*	
Pain in the past year	14.250	2.543–79.849	**0.003**	
Frequency of sugary drinks	3.994	1.357–11.753	**0.012**	
Presence of asthma	1.345	0.457–3.958	0.590	
B. Binary logistic regression—Low salivary flow < 1.0 mL/min (EPV = 12.7; Nagelkerke R^2^ = 0.277; Hosmer–Lemeshow *p* = 0.278)				
Variable	OR	95% CI	*p*	
Pain in the past year	10.907	1.230–96.746	**0.032**	
Frequency of sugary drinks	0.714	0.301–1.694	0.445	
Presence of asthma	4.017	1.443–11.178	**0.008**	
C. Linear regression—SOHO-5 Child score (F(3,85) = 5.633, *p* = 0.001; R^2^ = 0.166)				
Variable	B	95% CI	β	*p*
Pain in the past year	1.583	0.473 to 2.693	0.308	**0.006**
Frequency of sugary drinks	−0.069	−0.769 to 0.631	−0.020	0.845
Presence of asthma	0.732	−0.116 to 1.581	0.184	0.090
D. Linear regression—SOHO-5 Total score (F(3,85) = 15.888, *p* < 0.001; R^2^ = 0.359)				
Variable	B	95% CI	β	*p*
Pain in the past year	4.970	3.266 to 6.673	0.552	**<0.001**
Frequency of sugary drinks	0.081	−0.993 to 1.154	0.013	0.882
Presence of asthma	0.693	−0.609 to 1.996	0.099	0.293

*p* values in **bold** are statistically significant. OR: odds ratio; CI: confidence interval; B: unstandardized coefficient; β: standardized coefficient; EPV: events per variable.

## Data Availability

The data presented in this study are not publicly available due to ethical restrictions and privacy considerations related to the participation of minor subjects. Data may be available upon reasonable request to the corresponding author (S.V.M.), subject to ethical approval by the relevant institutional committees.
